# The Reliability of Foot and Ankle Bone and Joint Kinematics Measured With Biplanar Videoradiography and Manual Scientific Rotoscoping

**DOI:** 10.3389/fbioe.2020.00106

**Published:** 2020-03-10

**Authors:** Jayishni N. Maharaj, Sarah Kessler, Michael J. Rainbow, Susan E. D’Andrea, Nicolai Konow, Luke A. Kelly, Glen A. Lichtwark

**Affiliations:** ^1^School of Human Movement and Nutrition Sciences, The University of Queensland, Brisbane, QLD, Australia; ^2^Department of Mechanical and Materials Engineering, Queen’s University, Kingston, ON, Canada; ^3^Department of Orthopaedics, Brown University, Providence, RI, United States; ^4^Department of Kinesiology, The University of Rhode Island, Kingston, RI, United States; ^5^Department of Biological Science, University of Massachusetts, Lowell, MA, United States

**Keywords:** motion capture, foot, gait analysis, *in vivo*, intra-operator reliability, inter-operator reliability

## Abstract

The intricate motion of the small bones of the feet are critical for its diverse function. Accurately measuring the 3-dimensional (3D) motion of these bones has attracted much attention over the years and until recently, was limited to invasive techniques or quantification of functional segments using multi-segment foot models. Biplanar videoradiography and model-based scientific rotoscoping offers an exciting alternative that allows us to focus on the intricate motion of individual bones in the foot. However, scientific rotoscoping, the process of rotating and translating a 3D bone model so that it aligns with the captured x-ray images, is either semi- or completely manual and it is unknown how much human error affects tracking results. Thus, the aim of this study was to quantify the inter- and intra-operator reliability of manually rotoscoping *in vivo* bone motion of the tibia, talus, and calcaneus during running. Three-dimensional CT bone volumes and high-speed biplanar videoradiography images of the foot were acquired on six participants. The six-degree-of-freedom motions of the tibia, talus, and calcaneus were determined using a manual markerless registration algorithm. Two operators performed the tracking, and additionally, the first operator re-tracked all bones, to test for intra-operator effects. Mean RMS errors were 1.86 mm and 1.90° for intra-operator comparisons and 2.30 mm and 2.60° for inter-operator comparisons across all bones and planes. The moderate to strong similarity values indicate that tracking bones and joint kinematics between sessions and operators is reliable for running. These errors are likely acceptable for defining gross joint angles. However, this magnitude of error may limit the capacity to perform advanced analyses of joint interactions, particularly those that require precise (sub-millimeter) estimates of bone position and orientation. Optimizing the view and image quality of the biplanar videoradiography system as well as the automated tracking algorithms for rotoscoping bones in the foot are required to reduce these errors and the time burden associated with the manual processing.

## Introduction

Approximately 25% of the bones in our body are contained within our feet and the complex interaction between these bones is important for our capacity to walk and run (Hicks, 1954; Ker et al., 1987). However, due to the large number of small bones and the multi-articular structure of the foot, it is difficult to accurately measure bone motion to inform our understanding of foot function during locomotion. To better understand the function of the human foot in health and disease, accurate measurements of foot bone motion are needed.

Current methods to quantify foot motion are limited in both applicability and resolution. Bone-pin studies have provided insight into foot bone function during locomotion (Arndt et al., 2007; Nester et al., 2007; Lundgren et al., 2008). However, this approach is invasive, carries a risk of infection and is likely to be unsuitable for use in clinical populations. Soft tissue movement can often also cause pin bending, and thus may cause errors in kinematics. Optical motion capture has also informed the understanding of human foot function via the implementation of multi-segment foot models (Carson et al., 2001; Jenkyn and Nicol, 2007; Leardini et al., 2007; Rankine et al., 2008; Oosterwaal et al., 2016). This approach does not carry the risks of bone-pin approaches and can be implemented in clinical populations (Leardini et al., 2019). However, information obtained from multi-segment foot models is relatively low in resolution, as researchers are constrained to quantifying motion between functional segments, rather than anatomical joints (Nester et al., 2007).

Biplanar videoradiography and scientific rotoscoping are emerging as a non-invasive alternative approach to quantify three-dimensional (3D) motion of individual bones in the foot. This approach combines high-speed x-ray image sequences with 3D bone volumes obtained from computed tomography (CT) scans to track 3D bone movement (You et al., 2001; Miranda et al., 2011, 2013). Biplanar videoradiography has provided valuable insights into the function of the wrist (Akhbari et al., 2019), shoulder (Bey et al., 2006, 2008), hip (Dimitriou et al., 2015), and knee (Akbarshahi et al., 2010; Miranda et al., 2013) during dynamic tasks. Recently, it has also been shown to accurately capture 3D skeletal motion of the bones in the foot (Ito, 2015; Wang et al., 2015) and subsequently used to calculate their foot kinematics (Roach et al., 2016, 2017; Kessler et al., 2019).

Most of the current model-based algorithms of biplanar videoradiography data require manual intervention, where a 3D bone volume is rotated and translated until its 2D projection is aligned with two calibrated x-ray images. This process has been labeled scientific rotoscoping (Gatesy et al., 2010). These ‘hand tracked’ poses can then either be used as initial guesses to further optimize the kinematics or they can be analyzed directly. Scientific rotoscoping is accurate for tracking the isolated tibia, talus, and calcanei bones (Wang et al., 2015) but the reliability of the method remains to be determined for tracking bone movement during gait when bones are occluded at times. Presently, it is unknown how well this approach applies to the foot, where the bones are relatively small in size with irregular geometries and narrow joint spaces and x-ray images contain many overlapping bones. Given the requirements for manual alignment, the complex anatomy of the foot, and the ability to use many different software settings during the tracking process, there is potential for human measurement error and bias to be introduced when different operators track data, or when a single operator tracks data across multiple sessions.

Therefore, the aim of this study was to test the repeatability (intra-operator reliability) and reproducibility (inter- operator reliability) of manually rotoscoping the tibia, talus, and calcaneus across the stance phase of running, without controlling for user preferences. We hypothesized that tracking poses (position and orientation) of bones across the stance phase would be highly repeatable (intra-operator reliability) across different sessions and moderately reproducible (inter-operator reliability) between different operators. Further, we hypothesized that the calculated joint kinematics would be less reliable than the tracked bone orientations, as errors were likely to be amplified when calculating motion between bones across the stance phase.

## Materials and Methods

Six healthy participants (three males, three females) with no history of musculoskeletal injuries in the previous 6 months participated in the study. Mean (± standard deviation) subject height and body mass were 174 ± 8 cm, and 77 ± 13 kg respectively. Participants gave written consent to partake in the study at W.M. Keck Laboratory, Brown University. The experimental protocol was approved by The University of Queensland and the Providence VA Medical Center Ethics Committee and conducted according to the Declaration of Helsinki. This data set has been used in a previous publication, see Kessler et al. (2019).

### Biplanar Videoradiography System

**FIGURE 1:**
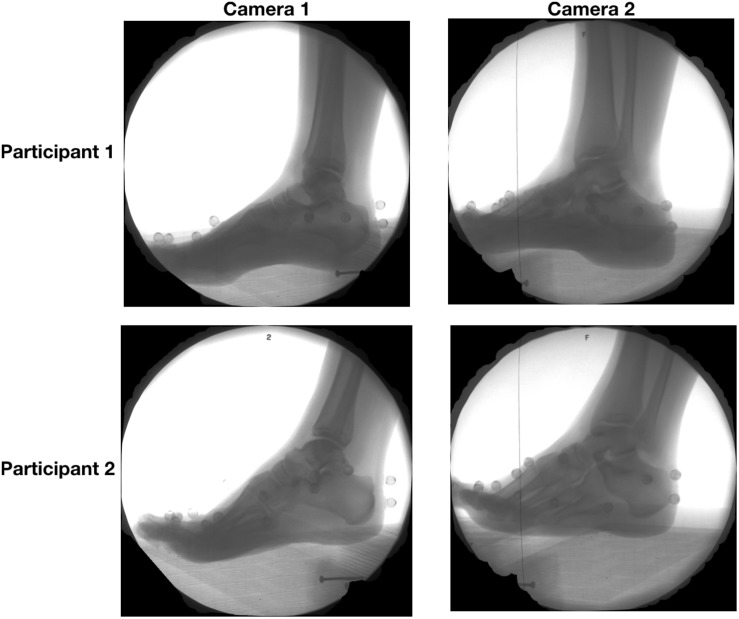
Radiographic images captured from camera 1 **(left)** and camera 2 **(right)** of two sample participants during the stance the phase of running.

Participants ran at a self-selected pace along a 10 m walkway while high-speed biplanar videoradiography was used to record x-ray images of the right foot (Figure 1) at 250 Hz. The biplanar videoradiography system, has been previously validated by Brainerd et al. (2010) and Miranda et al. (2011). The biplanar videoradiography system consisted of two x-ray generators (EMD Technologies CPX 3100CV) and two image intensifiers (Shimadzu Medical Systems, model AI5765HVP), optically coupled to synchronized high-speed video cameras (Phantom IV, Vision Research). The system was configured with a 130° inter-beam angle, a source-image-distance of 1.59 and 1.61 m for the two intensifiers, and object-to-intensifier distance of 0.75 m. Radiographic images were acquired with the x-ray generators in continuous radiographic mode (79 kVp, 100 mA, 2000 ms exposure time). Image resolution for each x-ray image was 1760 × 1760. All radiographic images were undistorted using an “un- distortion” grid (Brainerd et al., 2010) and calibrated using a custom calibration cube in XMALab software (XMALab, Brown University, Providence, RI, United States) (Knörlein et al., 2016). The calibration object was used to determine the location of the two high-speed cameras and x-ray sources.

### CT Scans

To create a 3D volume for each bone, a CT scan of the right foot of each participant was captured with the participant lying prone with the ankle in a plantarflexed orientation (average resolution: 0.4193 mm × 0.4193 mm × 0.625 mm). Two participants received CT scans at a different imaging location and thus had a slightly different average resolution (0.4883 mm × 0.4883 mm × 0.312 mm). The tibia, talus, and calcaneus of each subject was subsequently segmented from other bones and soft tissues using Mimics17 (Materialise, Leuven, Belgium), to provide 3D tessalated surface mesh and ‘partial volume’ of the bones. Briefly, the partial volume is a term used to describe the CT volume, it includes the original grayscale intensity values of the bone of interest with the voxels corresponding to other structures set to black.

### Data Processing

The position and orientation of each bone was determined via scientific rotoscoping using the open source software package, Autoscoper (Brainerd et al., 2010) (Autoscoper V2, Brown University, Providence, RI, United States), as previously detailed in Kessler et al. (2019). In brief, for each frame, the partial volume of the bone of interest is used to create a digitally reconstructed radiograph (DRR) that is projected onto the two calibrated x-ray images. Contrast and edge detection filters were applied to the DRRs and the X-ray images, which were based on the operator’s preference for each bone and participant. The operator then rotated and translated the bone volume until the two DRRs (one for each x-ray image) matched the captured x-ray images. Every 10 frames of data were manually tracked for each trial. After the initial manual alignment, the operators, at their discretion, optimized the position and orientation of the bone by using the normalized cross-correlation between the DRRs and the x-ray images in both views and a downhill simplex algorithm. This local optimization algorithm was not developed for the bones in the feet and often resulted in an unfavorable output, and the optimized position was therefore not always used. No global optimization algorithms were used to track the bone motion. The remaining frames were aligned using a quaternion spline interpolation, which was used to interpolate between key frames. If the DRRs did not match intermediate frames between the key frames, a bisecting keyframe was chosen and the spline was updated. Greater manual adjustments (keyframes) were generally required during heel strike and propulsion, due to increased bone movement. For each bone for each participant, typically 20 key frames were manually tracked by each operator. All tracking was performed on one bone at a time for as much of the stance phase that the bone was visible in both cameras. To ensure adjacent bones were not colliding, all bones within a trial were animated together and visualized by the two operators in an iterative manner. The quality of the tracking was subsequently assessed by two investigators who were not involved in tracking data (LK and GL). Bone tracking was performed by two operators; both operators had prior experience tracking foot bones during locomotion. The first operator re-tracked all bones after a 2 month interval, to test intra-operator effects.

### Data Analysis and Statistics

**FIGURE 2:**
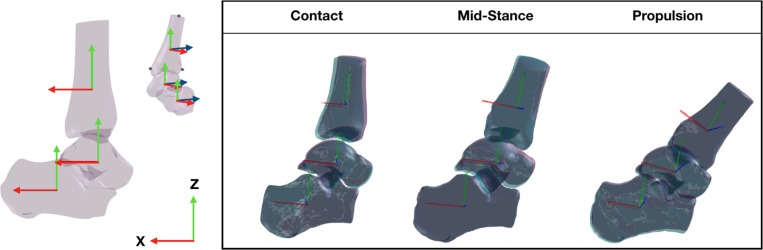
Three dimensional bone model of the tibia, talus, and calcaneus and their tracking solutions processed across two sessions, session 1 (purple bones) and session 2 (aqua bones) tracked by the same operator. The local coordinate system of each bone illustrated by the solid red (x-axis), blue (y-axis), and green (z-axis) lines were created using virtual markers on the medial malleolus, lateral and superior most aspect of the tbia and transformed across the gait cycle according to the tracking solution.

The position and orientation (pose) of each of the bones was expressed in a global coordinate system. To ensure the pose of each bone was anatomically relevant throughout the trial, the global coordinate system was defined by the orientation of the anatomical coordinate system of the tibia in a standing trial, when the foot is in a neutral position. The anatomical coordinate system of the tibia was defined by digitizing the tibia bone surface model. The Y-axis (medio-lateral) was defined by the vector between the medial malleolus and the most lateral aspect of the tibia; the Z-axis (superior-inferior) was defined between the most lateral aspect and the most superior aspect of the tibia; the X-axis (anterior–posterior) was defined orthogonal to the Y- and Z-axes (Figure 2). The global coordinate system during the standing trial was then assigned to each of the bone’s local coordinate systems such that the Euler angles of each of the bones during the standing trial were set to zero. The origin of each bone coordinate system was placed at the centroid of the bone surface mesh. The centroid of the tibia was calculated based on the proportion of the tibia captured on the CT scan, which was similar across the participants. The assignment of the global co-ordinate system during the standing trial was performed by a single operator (JM) and applied to the tracking undertaken by all operators, such that a comparison of the bone orientation and translation could be made based entirely on the ability to manually track the bones.

Noise was removed from the raw orientations (represented as quaternions) using spherical linear interpolation and from the raw positions using a 6 Hz fourth-order, low-pass Butterworth filter.

Joint angles (talus relative to the tibia [talus-tibia], calcaneus relative to the tibia [calcaneus-tibia], calcaneus relative to the talus [calcaneus-talus]) were computed from the filtered transformation matrixes and converted to Euler angles for graphical representation and statistical analysis. Tracking of both operators were aligned such that tracking commenced and ended at the same frames. The translations of each participant were normalized to the mean translation across the stride to enable comparable visualization (removing offsets due to foot placement differences across individual trials).

The similarity between bone translations, bone orientations and joint angles were compared using linear fit modeling (LFM) (Iosa et al., 2014) and root mean square (RMS) errors across coordinates planes. LFM was implemented to compare the variation in the time-series data across different tracking sessions (intra-operator), or trackers (inter-operator). The LFM has the advantage of assessing the variance across the entire stride cycle, providing indices of the strength of the linear relation (*R*^2^), amplitude agreement (a1) and offset in magnitude (a0) for each stride assessed. A perfect agreement is illustrated by an *R*^2^ and a1 equal to 1 and an a0 and RMSE equal to 0. The strength of the similarity across the entire waveform was determined by the *R*^2^ score, no relationship = 0.0 to 0.3, weak = 0.3 to 0.5, moderate = 0.5 to 0.7, and strong = 0.7 to 1.0 (Mukaka, 2012).

## Results

### Bone Translations

#### Intra-Operator Reliability

**FIGURE 3:**
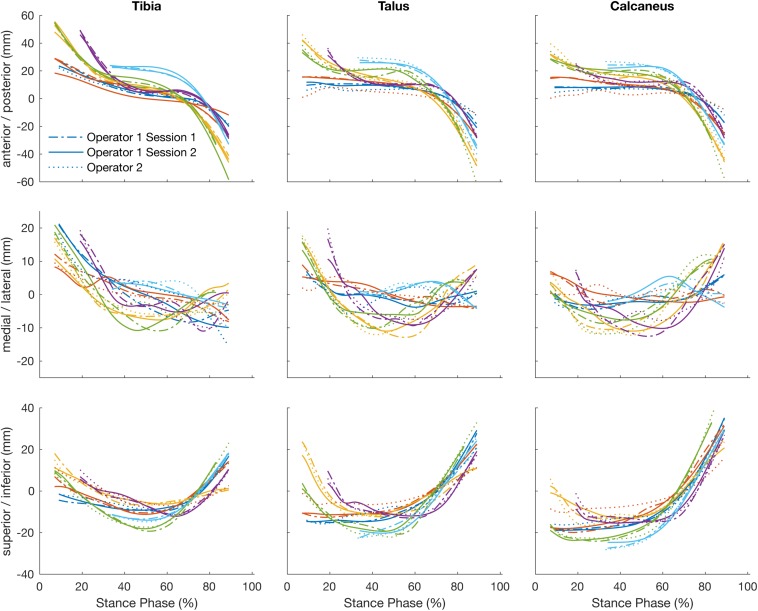
Plots illustrate time normalized translation of the tibia, talus, and calcaneus of each participant tracked by a single operator over two sessions, session 1 (dashed line) and session 2 (solid line) and operator two (dotted line).

The position of the tibia, talus, and calcaneus tracked by a single operator over two sessions and its reliability scores are illustrated in Figure 3 and Table 1. The strength of the linear relationship (*R*^2^) between the tracked positions across all planes was 0.94 (range: 0.80 to 0.99). The tracked bone positions along the X- and Z-axes (anterior/posterior and superior/inferior translations respectively) demonstrated the strongest linear relationship (*R*^2^ ∼ 0.99), followed by the Y-axis (medio/lateral translation, *R*^2^ = 0.85). The agreement in their amplitude (a1) was around the ideal value of 1 (range: 0.87 to 1.06) and the offsets in their magnitude (a0) varied randomly from −2.89 to −1.59 mm between planes. The mean RMS error calculated across all planes was 1.86 mm, ranging from 1.38 to 2.64 mm across all bones.

**TABLE 1:** Intra- and inter- operator reliability scores for bone translations.

	Linear fit model			
	R^2^	a1	a0 (mm)	RMS errors (mm)

		Intra-Operator		
**x - axis (anterior / posterior)**				
tibia	1(0.99−1)	1.01(0.99−1.03)	−1.7(−6.43−1.02)	1.44(0.48−2.26)
talus	0.99(0.99−1)	1.01(0.98−1.03)	−2.03(−6.48−2.6)	1.87(0.51−3.26)
calcaneus	0.99(0.98−1)	1.01(0.99−1.05)	−1.59(−3.93−0.82)	1.84(0.91−2.94)
**y - axis (medial / lateral)**				
tibia	0.86(0.74−0.91)	1.04(0.87−1.17)	−1.87(−6.37−2.51)	2.64(1.08−3.94)
talus	0.9(0.79−0.97)	1.05(0.9−1.22)	−2.89(−7.83−1.25)	1.7(0.76−2.44)
calcaneus	0.8(0.51−0.95)	0.87(0.45−1.04)	−1.67(−4.46−2.73)	2.09(0.96−3.12)
**z - axis (superior / inferior)**				
tibia	0.98(0.92−1)	1.06(0.97−1.29)	−2.23(−7.47−2.9)	1.38(0.59−2.42)
talus	0.98(0.93−1)	1.02(0.92−1.17)	−2.85(−8.29−5.27)	2.19(0.76−3.35)
calcaneus	0.99(0.99−1)	1.02(0.96−1.11)	−2.15(−5.6−2.42)	1.58(0.82−2.24)

		**Inter-Operator**		

**x - axis (anterior / posterior)**				
tibia	0.99(0.99−1)	0.97(0.93−1.03)	0.55(−2.97−4.36)	2.09(1.16−2.97)
talus	0.98(0.94−1)	0.99(0.94−1.06)	1.41(−6.68−7.62)	2.24(1.36−3.42)
calcaneus	0.97(0.86−1)	0.98(0.97−1.01)	2.33(−7.1−8.35)	2.65(0.84−4.68)
**y - axis (medial / lateral)**				
tibia	0.76(0.59−0.85)	0.79(0.52−1.08)	−0.32(−5.29−4.4)	3.32(1.65−4.96)
talus	0.75(0.07−0.97)	0.81(0.22−1.07)	1.22(−3.55−9.6)	2.15(1.42−3.08)
calcaneus	0.71(0.14−0.95)	0.86(0.64−1.08)	1.59(−5.88−7.18)	2.68(1−3.99)
**z - axis (superior / inferior)**				
tibia	0.97(0.94−0.99)	0.93(0.81−0.99)	2.91(−1.4−5.26)	1.55(1.24−1.76)
talus	0.98(0.96−1)	0.95(0.81−1.02)	2.45(−2.78−6.43)	1.86(1.15−2.59)
calcaneus	0.98(0.94−1)	0.97(0.86−1.02)	4.3(−1.93−10.35)	2.04(0.83−3.82)

*The mean strength of the linear fit model (R^2^) for all planes were 0.94 across sessions and 0.90 between operators, while the mean root mean square errors (RMS) were 1.86 mm between sessions and 2.29 mm between operators.*

#### Inter-Operator Reliability

The strength of the linear relationship between the tracked positions across operators (*R*^2^) was 0.90 (range: 0.71 to 0.99). The tracked bone positions along the X- and Z- axes (anterior/posterior and superior/inferior translations respectively) demonstrated the strongest linear relationship (*R*^2^ = 0.98 and 0.98), followed by the Y- axis (medio/lateral translation, *R*^2^ = 0.74). The agreement in their amplitude (a1) ranged form 0.79 to 0.99 and the offsets fluctuated randomly ranging from −0.32 to 4.30 mm between planes. The mean RMS error calculated across all planes was 2.29 mm, ranging from 1.55 to 3.32 mm across all bones.

### Bone Orientations

#### Intra-Operator Reliability

**FIGURE 4:**
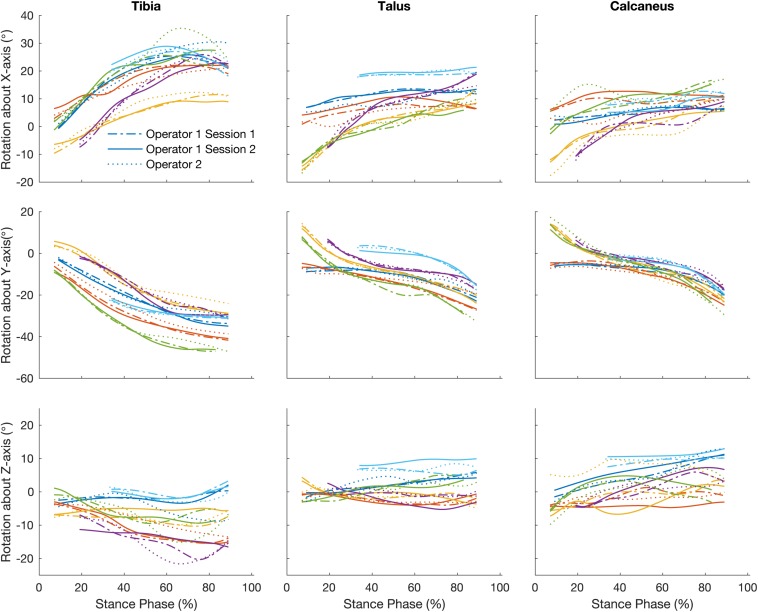
Traces illustrate time normalized oreintation of tibia, talus, and calcaneus of each participant tracked by a single operator over two sessions, session 1 (dash line) and session 2 (solid line) and operator two (dotted line).

Figure 4 illustrates the orientation of the tibia, talus, and calcaneus tracked by a single operator over two sessions and Table 2 reports its reliability scores. The strength of the linear relationship between the tracked bone orientations was 0.81 (0.54 to 0.99). The strongest linear relationship was found for bone orientations tracked about the Y-axis (0.99) followed by the X- and Z- axes (0.87 and 0.58 respectively). The agreement in their amplitude (a1) fluctuated between 0.65 and 1.66 and no biases in direction were found as illustrated by the range in offsets (−0.94 to 1.97). The mean RMS error calculated across all bones was 1.90°, ranging from 1.31 to 2.67°.

**TABLE 2:** Intra- and inter- operator reliability scores for bone orientations.

	Linear fit model			
	R^2^	a1	a0 (°)	RMS errors (mm)

		Intra-Operator		
**rotation about x - axis**				
tibia	0.94(0.87−0.98)	1.07(0.81−1.22)	0.96(−1.46−3.77)	2.29(1.84−2.73)
talus	0.88(0.76−0.98)	0.92(0.34−1.29)	0.4(−2.56−2.9)	1.86(1−2.89)
calcaneus	0.78(0.25−0.94)	0.8(0.43−1.18)	0.21(−2.9−2.14)	2.23(0.95−3.04)
**rotation about y - axis**				
tibia	0.99(0.95−1)	0.98(0.9−1.09)	−0.42(−1.63−1.1)	1.31(0.7−2.53)
talus	0.98(0.98−1)	0.98(0.86−1.09)	−0.63(−3.24−1.39)	1.52(0.72−2.12)
calcaneus	0.99(0.97−0.99)	1(0.95−1.11)	−0.93(−3.2−2.36)	1.42(0.9−1.92)
**rotation about z - axis**				
tibia	0.63(0.09−0.97)	0.86(0.26−1.35)	−0.94(−4.68−0.67)	2.01(0.83−3.07)
talus	0.54(0.01−0.88)	0.65(−0.11−1.25)	−0.17(−2.75−1.46)	1.74(0.78−3.02)
calcaneus	0.56(0.03−0.97)	0.73(0.24−1.26)	0.73(−1.36−3.08)	2.67(1.92−3.25)

		**Inter-Operator**		

**rotation about x - axis**				
tibia	0.89(0.7−0.98)	0.83(0.71−0.99)	1.18(−1.75−6.8)	2.87(1.24−4.88)
talus	0.74(0.13−0.97)	0.74(0.35−1.33)	−0.03(−4.8−2.51)	2.4(1.19−3.24)
calcaneus	0.63(0.1−0.96)	0.95(0.2−1.42)	1.93(−0.69−4.84)	3.5(1.16−6.56)
**rotation about y - axis**				
tibia	0.97(0.93−1)	1.09(0.89−1.24)	0.88(−0.42−1.94)	2.31(0.88−3.73)
talus	0.99(0.97−1)	1.04(0.87−1.33)	0.08(−2.06−2.41)	1.35(0.91−1.72)
calcaneus	0.99(0.96−1)	1.03(0.87−1.33)	1.1(−0.2−3.25)	1.96(0.58−2.99)
**rotation about z - axis**				
tibia	0.45(0.01−0.98)	0.45(0.08−1.17)	−0.49(−4.76−2.08)	3.01(1.53−4.66)
talus	0.61(0.18−0.8)	0.91(0.47−1.59)	−1.89(−4.61−1.19)	1.85(0.68−2.99)
calcaneus	0.46(0.17−0.93)	0.76(−0.58−2.4)	1.31(−2.09−6.13)	4.12(2.1−10.29)

*The strength of the linear fit model (R^2^) for bone oreintaions across all planes was 0.81, with a mean root mean square (RMS) error of 1.90° when a single operator tracked the orientations of bones across multiple sessions. Across operators the mean R^2^ and RMS error were 0.75 and 2.60° respectively.*

#### Inter-Operator Reliability

The strength of the linear relationship for bone orientations trascked by different operators was 0.75 (0.45 to 0.99). Similar to the intra-operator scores orientations about the Y-axis demonstrated the greatest similarity (0.98), followed by the X- and Z- axes (0.75 and 0.50 respectively). The agreement in their amplitude (a1) fluctuated between 0.45 and 1.09. The offsets in magnitude fluctuated randomly from −1.89 to 1.93°. The mean RMS error calculated across all bones was 2.60° (range: 1.35 to 4.11°).

### Joint Angles

#### Intra-Operator Reliability

**FIGURE 5:**
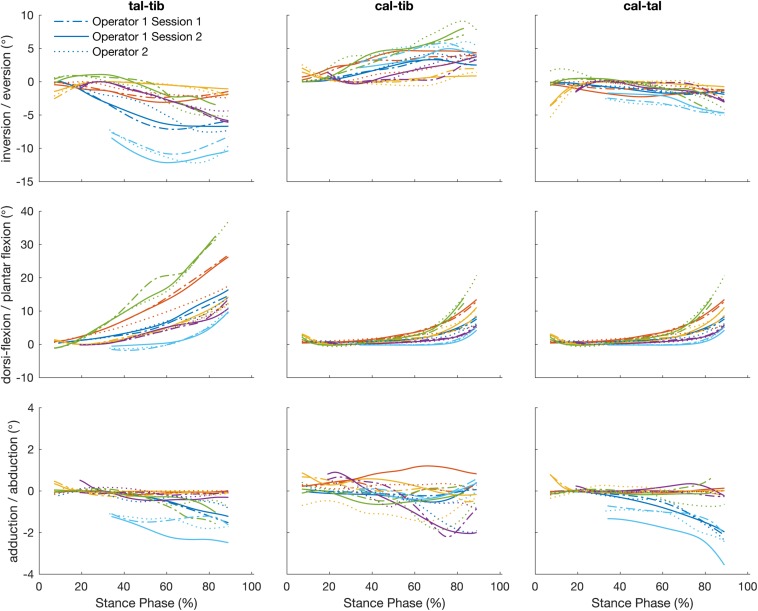
Plots illustrate time normalized kinematics of the talus relative to the tibia (talus-tibia) and calcaneus relative to the tibia (calcaneus-tibia) and talus (calcaneus -talus) of each participant tracked by a single operator over two sessions, session 1 (dash line) and session 2 (solid line) and operator two (dotted line).

The angles calculated between the talus-tibia, calcaneus-tibia, calcaneus-talus tracked by a single operator over two sessions demonstrated in Figure 5 had a linear strength of 0.83 (0.59 to 0.99). The strongest linear relationship was found for the plantar/dorsi-flexion joint angles (0.99), followed by inversion/eversion (0.85) and abduction/adduction (0.65). The offsets in magnitude were not biased to a particular direction and ranged from −0.51 to 0.15° in all axes of measurement. The agreement in amplitude (a1) fluctuated between 0.52 and 1.24 (Table 3). The mean RMS error calculated across planes was 0.67°, ranging from 0.29 to 1.12°.

**TABLE 3:** Reliability scores for joint kinematics: motion of the talus relative to the tibia (talus-tibia) and calcaneus relative to the tibia (calcaneus-tibia) and talus (calcaneus -talus).

	Linear fit model			
	R^2^	a1	a0 (°)	RMS errors (mm)

		Intra-Operator		
**inversion / eversion**				
tibia	0.91(0.79−1)	1.24(0.86−2.02)	−0.06(−1.41−0.99)	0.79(0.18−1.58)
talus	0.84(0.66−0.96)	1.05(0.8−1.88)	0.05(−1.01−0.48)	0.71(0.24−0.97)
calcaneus	0.82(0.51−0.95)	0.98(0.43−1.67)	−0.36(−0.92−0.45)	0.75(0.25−1.21)
**dorsi - / plantar flexion**				
tibia	0.99(0.98−1)	1(0.85−1.2)	0.15(−0.58−0.88)	1.12(0.56−1.81)
talus	0.99(0.99−1)	1(0.92−1.09)	−0.04(−0.59−0.43)	0.83(0.54−1.38)
calcaneus	1(0.99−1)	0.93(0.77−1)	−0.51(−1.8−0.21)	0.69(0.27−1.51)
**adduction / abduction**				
tibia	0.59(0.08−0.99)	0.79(0.05−1.49)	0(−0.09−0.09)	0.29(0.03−0.73)
talus	0.65(0.4−0.96)	0.52(−0.47−0.99)	0.05(−0.15−0.34)	0.42(0.16−0.78)
calcaneus	0.72(0.07−0.99)	0.91(0.1−2.43)	0.03(−0.09−0.19)	0.4(0.07−1.24)

		**Inter-Operator**		

**inversion / eversion**				
tibia	0.88(0.79−0.94)	0.8(0.51−1.28)	−0.24(−1.39−1.13)	0.89(0.48−1.26)
talus	0.79(0.57−0.99)	0.76(0.36−0.98)	0.21(−0.71−1.45)	0.97(0.5−1.81)
calcaneus	0.8(0.24−0.96)	0.89(0.54−1.37)	0.01(−0.91−1)	0.71(0.21−1.14)
**dorsi - / plantar flexion**				
tibia	0.99(0.98−1)	1.03(0.86−1.16)	0.04(−0.88−0.69)	0.93(0.58−1.24)
talus	0.99(0.97−1)	1.06(0.97−1.25)	0.13(−0.87−1.21)	1.02(0.77−1.66)
calcaneus	1(0.99−1)	1.06(0.89−1.26)	0.52(−0.22−2.28)	0.6(0.16−0.77)
**adduction / abduction**				
tibia	0.49(0.07−0.87)	0.84(−0.34−1.61)	−0.31(−0.7−0.01)	0.32(0.09−0.67)
talus	0.25(0−0.74)	0.47(−0.15−2.21)	0.11(−0.72−0.57)	0.79(0.29−1.24)
calcaneus	0.57(0.07−0.95)	0.57(−1.04−1.68)	−0.11(−0.49−0.21)	0.42(0.06−1.11)

*The R^2^ value from the linear fit model and the root mean square (RMS) error was 0.83 and 0.67° when a single operator tracked the orientations of bones across multiple sessions. The R^2^ values for kinematics calculated between operators was 0.75 and the mean RMS error range was 0.74°.*

#### Inter-Operator Reliability

The strength of the linear relationship for the angles calculated by the tracking of different operators was 0.75 (0.25 to 0.99). Similar to the intra-operator scores, greater similarity was shown for plantar/dorsi- flexion (0.99), followed by the inversion/eversions (0.82) and abduction/adduction (0.44). The offsets in magnitude ranged from −0.31 to 0.52° and their amplitude agreement fluctuated between 0.47 and 1.06. The mean RMS error calculated across planes was 0.74° (range: 0.32 to 1.02°).

## Discussion

Biplanar videoradiography along with scientific rotoscoping is a promising, non-invasive method that has the potential to provide unique insight into the function of individual joints of the foot, in healthy as well as clinical populations. We report that model-based scientific rotoscoping of biplanar videoradiography data provides repeatable and reproducible measures of foot bone position, orientation and joint kinematics during locomotion. These results are not a reflection of the accuracy of capturing bones in the foot using biplanar videoradiography (Brainerd et al., 2010). Our repeatability (intra-operator) results indicate that across sessions, a single operator is able to track the translations and orientations of the tibia, talus, and calcaneus with a mean RMS error of approximately 1.86 mm and 1.90° respectively. Across operators the RMS error range was approximately 2.3 mm and 2.6° about all three planes of motion. Bone orientations and joint angle waveforms were most reliable (average *R*^2^ > 0.9, average a1 ≈ 1 and average a0 ≈ 0) in the sagital plane (plantar and dorsi- flexion for joint rotations), followed by the frontal plane (inversion and eversion for joint rotations). The transverse plane (abduction and adduction for joint rotations) had the weakest reliability results (average *R*^2^ < 0.7, most highly variable a1 and a0). While a greater range of error is apparent in the absolute positions and orientations of the bones quantified across operators, our results indicate that the gross mechanical behavior of the bones is reproducible.

The variance observed across sessions and operators was larger than the accuracy reported for the same bones when using cadaveric specimens (Ito, 2015; Wang et al., 2015). This may be due to a variety of experimental and measurement factors, including the magnitude and the velocity of bone motion and overlap of surrounding bones (Bey et al., 2008; Miranda et al., 2011). The combined field of view of the two x-ray systems was at times too small to capture all foot bones across the entire stance phase, requiring the operator to speculate about the pose of the bone based on other visible edge definitions. This was most common for the calcaneus, which also demonstrated the largest range in RMS errors between operators. Improving the location of heel contact within the visualization volume of the biplanar videoradiography system may reduce such errors, particularly as changing the size of the field of view may be difficult. Additionally, the location and angle of the x-ray generators and intensifiers may have contributed to decreased reliability, particularly in out-of-plane motion. The image intensifiers were oriented primarily in the sagittal plane relative to the tibia, and this affects the ability to visualize and track bones in the frontal and transverse planes. Better visibility of the lateral aspect of the foot would improve the alignment of the bone and enhance the out-of-plane reliability. More work can be done to further optimize imaging planes to make the tracking task more reliable. Finally, errors may be mitigated through the development of more robust global optimization algorithms for registration, estimation, and ultimately reliability of bone pose estimation. The implementation of automated tracking algorithms, with improved algorithms for calculating global optimal solutions (e.g., particle swarm optimization algorithms; Akhbari et al., 2019) tailored to the foot bones, potentially with multi-bone approaches, may further improve the reliability of biplanar data processing.

How does operator-dependent variability compare to between subject (natural) variation? Figures 3–5 illustrate qualitatively that the variation across subjects is much larger than the differences within and across operators due to the scientific rotoscoping process. The limited variation within and among operators suggests that scientific rotoscoping data can be used to explain factors that contribute to subject-specific joint motion. This finding is important as there is a large amount of variation in individual bone shapes, soft tissue properties, and neuromechanics (Kelikian and Sarrafian, 2011) and this variation may contribute to the development of pathology.

The RMS error range for our joint angles was approximately 1° between sessions and operators across all bones. These ranges of differences seem to be sufficient for characterizing foot angles with high confidence, particularly compared to measures of external segment tracking and optical motion capture. Using a six-degree-of-freedom kinematic foot model, an absolute difference of 3–6°, depending on the segment, is reported for inter-session reliability of foot kinematics (Caravaggi et al., 2011). These reliability values may not be comparable to those measured in this study as within operator reliability of optical motion capture requires operators to place markers consistently while in scientific rotoscoping the operator must track the same bones in a reliable manner. Nevertheless, the fact that scientific rotoscoping demonstrates improved reliability compared to optical motion capture is an encouraging sign that this technology may be useful for specific clinical or applied questions. Maximum error ranges of the intra- and inter-operator comparisons also suggest that the burden of manually tracking biplanar data may be spread across multiple operators and tracking sessions, particularly when quantifying individual bone translation, orientations and six-degree-of-freedom joint kinematics in Euler angles. Sharing this burden across well-trained operators will enhance the usability of this approach, particularly as it currently takes approximately 3 to 4 h to rotoscope a single bone. For a single running stride approximately 20 key frames need to be manually aligned, taking anywhere from 10 to 30 min per key frame. The intra- and inter-operator variance may, however, be too high for certain measures to be made from bone motion, such as the instantaneous helical axes, strains of soft tissues such as ligaments, fascia, or muscle, or joint contact measurements.

This is the first study to investigate the reliability of rotoscoping skeletal motion using bone models between sessions and operators during *in vivo* human locomotion. Previous studies have investigated the accuracy of the biplanar videoradiography hardware and the Autoscoper algorithm (Bey et al., 2006, 2008; Miranda et al., 2011), rather than the reliability of the rotoscoping process. These studies typically compare skeletal motion quantified using bone models (markerless tracking) to those tracked using the position of implanted tantalum beads (marker-based tracking) to illustrate the low systemic error associated with the technique. Our results are not comparable to the sub-millimeter and sub-degree accuracy reported in such studies (You et al., 2001; Miranda et al., 2011, 2013) and suggest that errors in the processing may emerge when rotoscoping in a manual manner. Our results are partly supported by the findings of Miranda et al. (2011), who reported larger markerless errors during static trials compared to dynamic protocols because of the initial guess required in markerless tracking.

While our measures are able to quantify the reliability and repeatability of tracking bones in the foot captured using biplanar videoradiography, they cannot reflect on its accuracy (Brainerd et al., 2010). Methods to quantify the validity of tracking skeletal motion are highly invasive and require surgical implantation of tantalum beads into individual bones. *Ex vivo* studies have performed such procedures and demonstrated that tracking bones in the foot using optimization techniques is highly accurate during dynamic gait movements, they report errors of 0.4 mm in translation and 0.4° in orientation (Ito, 2015). While implementing gait simulators to replicate human gait has several limitations and may not truly replicate the forces and motion that the biological tissues are exposed to, the results of these studies certainly indicate that methods of tracking bones in the foot are accurate. Furthermore, the radiation exposure to the participant, the small visualization volume of the x-ray beams and the large time burden of model-based rotoscoping are other potential limitations of this technique. However, provided the research question can be answered in the presence of precision errors of approximately 2°, the time burden may be spread across multiple operators and tracking sessions improving the feasibility of this approach in foot biomechanics research. The present results would suggest that measurements requiring more accurate measures of bone position and orientation (like joint contact measures), may require additional procedures to increase the accuracy and reliability, as mentioned previously.

Biplanar videoradiography and model-based scientific rotoscoping is a powerful tool that allows 3D measurement of individual foot bones during locomotion. We have shown that this method, which currently requires considerable manual tracking, is repeatable and reproducible. The kinematics subsequently calculated from these measurements are also consistent. The ability to reliably reconstruct the skeletal kinematics of the human foot in 3D can provide critical new perspectives for understanding foot function and pathology. The applied methodologies of biplanar videoradiography may also help untangle the relationship between anatomical features of foot bones and the joint motions that result from their articulations. They may also serve in validating commonly implemented multi-segment foot models (Kessler et al., 2019).

## Data Availability Statement

The dataset is available upon request from MR.

## Ethics Statement

The studies involving human participants were reviewed and approved by The University of Queensland and the Providence VA Medical Center Ethics Committee. The patients/participants provided their written informed consent to participate in this study.

## Author Contributions

GL, LK, NK, SD’A, and MR contributed to the conception, design of the study, as well as the data acquisition. JM, SK, LK, MR, and GL organized the database, while JM and SK performed the scientific rotoscoping of the bones. JM and GL were responsible for the data analysis and statistical testing. All authors contributed to the manuscript revision, read, and approved the submitted version.

## Conflict of Interest

One or more of the authors (GL, LK) has received funding from the UQ Collaborative Industry Engagement Fund, collaborating with Asics Oceania. SD’A has received support and resources, as well as, the use of facilities at the Providence VA Medical Center, Providence, RI, United States. The remaining authors declare that the research was conducted in the absence of any commercial or financial relationships that could be construed as a potential conflict of interest. The reviewer, JC, declared a past supervisory role with one of the authors, SD’A, to the handling Editor.
